# Pain Perception During Transperineal and Transrectal Prostate Biopsy Under Local Anesthesia: a Prospective Analysis of a Multi-ethnic and Diverse Cohort

**DOI:** 10.1590/S1677-5538.IBJU.2025.0512

**Published:** 2026-01-28

**Authors:** Kevin Joseph Chua, Lorenzo Storino Ramacciotti, Masatomo Kaneko, Yuta Inoue, Luis Medina Navarro, Jie Cai, Manju Aron, Pierre Halteh, Eric Kau, Anne Schuckman, Sij Hemal, Mihir Desai, Hooman Djaladat, Inderbir S. Gill, Monish Aron, Andre Luis Abreu

**Affiliations:** 1 University of Southern California Keck School of Medicine USC Institute of Urology and Catherine & Joseph Aresty Department of Urology Los Angeles California USA USC Institute of Urology and Catherine & Joseph Aresty Department of Urology, Keck School of Medicine, University of Southern California, Los Angeles, California, USA; 2 USC Institute of Urology, Center for Image-Guided Surgery Focal Therapy and Artificial Intelligence for Prostate Cancer USA USC Institute of Urology, Center for Image-Guided Surgery, Focal Therapy and Artificial Intelligence for Prostate Cancer; 3 University of Southern California Departments of Pathology Keck School of Medicine Los Angeles California USA Departments of Pathology Keck School of Medicine, University of Southern California, Los Angeles, California, USA; 4 University of Southern California Departments of Radiology Keck School of Medicine Los Angeles California USA Departments of Radiology Keck School of Medicine, University of Southern California, Los Angeles, California, USA

**Keywords:** Prostatic Neoplasms, Race Factors, Socioeconomic Factors

## Abstract

**Purpose:**

To assess factors associated with patients’ self-assessed pain scores during prostate biopsy (PBx) performed exclusively under local anesthesia (LA).

**Materials and Methods:**

Consecutive patients who underwent MRI followed by a transperineal (TP) or transrectal (TR) PBx under LA were prospectively assessed. Race and ethnicity were self-reported according to NIH standards. Socioeconomic status was assessed using the Distressed Community Index (DCI). Pain was evaluated with a visual analog scale (0-10) after the procedure. Univariable and multivariable linear regression analyses were performed to correlate clinical parameters related to pain.

**Results:**

A total of 419 patients underwent TP (77%) or TR (23%) PBx. Overall, 14% of patients were Asian, 5% Black, 17% Latino, 12% Others, and 53% White. Of the cohort, 20% of Black and 27% of Latino patients were most distressed (DCI 80-100) compared with 4% of Asian, 9% of Other, and 5% of White patients (p<0.001). The median (IQR) self-assessed pain levels were higher for Black 5 (2-5) and Latino 4 (3-5) compared to Asian 3 (2-4), Other 3 (2-5), and White 3 (2-4) patients (p=0.01). On multivariable analysis, younger patients, Black or Latino patients, and the number of lesions on MRI were independent predictors for pain levels.

**Conclusions:**

PBx under LA alone are generally well tolerated; however, there is a subset of patients who experience more pain, including Black and Latino, younger patients, and those with more MRI suspicious lesions. Discussion of these pain risk factors is important for patients when choosing to have a biopsy performed under LA versus sedation.

## INTRODUCTION

The diagnosis of prostate cancer is usually made by a prostate biopsy (PBx), making this one of the most common procedures in Urology with more than 1 million PBx being performed in the US and Europe combined (1–4). While performing PBx in the operating room under sedation may decrease a patient's pain, it may increase the costs, risk of complications, and is subject to operating room availability. Although PBx under local anesthesia may be more practical, time-efficient and cost-effective, a proportion of patients may experience severe pain. Therefore, identifying predictors for pain during prostate biopsy may allow for improved prebiopsy counseling.

Several potential factors may influence pain perception during medical procedures including race/ethnicity and socioeconomic status (SES) (5, 6). Within an experimental setting, as demonstrated by Rahim Williams et al., lower pain tolerances were seen in African American and Hispanic populations (7). Additionally, Thurston et al had identified that lower SES was associated with increased postoperative pain (8). To the best of our knowledge, there is no study evaluating the impact of race/ethnicity and socioeconomic status on perception of pain during a prostate biopsy. The aim of this study is to identify different factors associated with pain during an in-office PBx exclusively under local anesthesia with the hypothesis that race/ethnicity, SES, age and other clinical and demographic parameters would affect pain levels and tolerance to PBx.

## MATERIALS AND METHODS

### Study Population

Consecutive patients who underwent multiparametric MRI (mpMRI) followed by transperineal (TP) or transrectal (TR) PBx between June 2020 and May 2023 were prospectively assessed (IRB #HS-13-00663). Exclusion criteria were I) mpMRI acquired more than 6 months prior to the PBx; II) mpMRI that did not meet Prostate Imaging Reporting & Data System (PIRADS) standards. III) prior treatment for prostate cancer IV) prior surgery for benign prostatic hyperplasia; V) Saturation PBx.

### MRI Acquisition and Interpretation

All mpMRIs, whether performed at an outside institution or our institution, were interpreted at our institution in accordance with the Prostate Imaging Reporting & Data System (PIRADS) v2.0 or v2.1 by radiologists with expertise in prostate mpMRI reading.(9) The index lesion location was assigned as base, mid or apex, and anterior or posterior according to PIRADS definition (9). If a PIRADS ≥ 3 lesion was traversing more than one of these areas, it was counted for both.

### Prostate Biopsy Protocol

All PBx were carried out transperineally or transrectally by a single urologist (ALA) using a three dimensional organ tracking elastic image fusion system (Trinity, Koelis®, Grenoble, France) and 18G needle biopsy as previously described (10–15). All patients underwent MRI followed by TP or TR 12-14 core systematic PBx, with a minimum of two additional target-PBx cores per PIRADS ≥ 3 lesion. The PBx specimens were evaluated by a uropathologist according to International Society of Urological Pathology (ISUP) guidelines (16). Clinically significant prostate cancer (CSPCa) was defined as Grade Group ≥2.

During the study period, the operator (ALA) was transitioning from TR to TP PBx. Once this transition was complete, TP biopsies became the preferred approach, while TR biopsies were performed solely based on patient preference. The operator had extensive experience with both biopsy techniques, having surpassed their respective learning curves. Patient characteristics, lesion location, imaging findings, or other individual factors did not influence the biopsy approach for each patient.

### Local Anesthesia Administration

All procedures were performed exclusively under local anesthesia, using techniques widely accepted as part of the current standard of care (17, 18). No additional analgesics, anxiolytics, or sedatives were used. Prior to the biopsies the patients were appropriately counseled about the procedure protocols and watched an educational and informative video

acess: LINK

**Figure 1:**
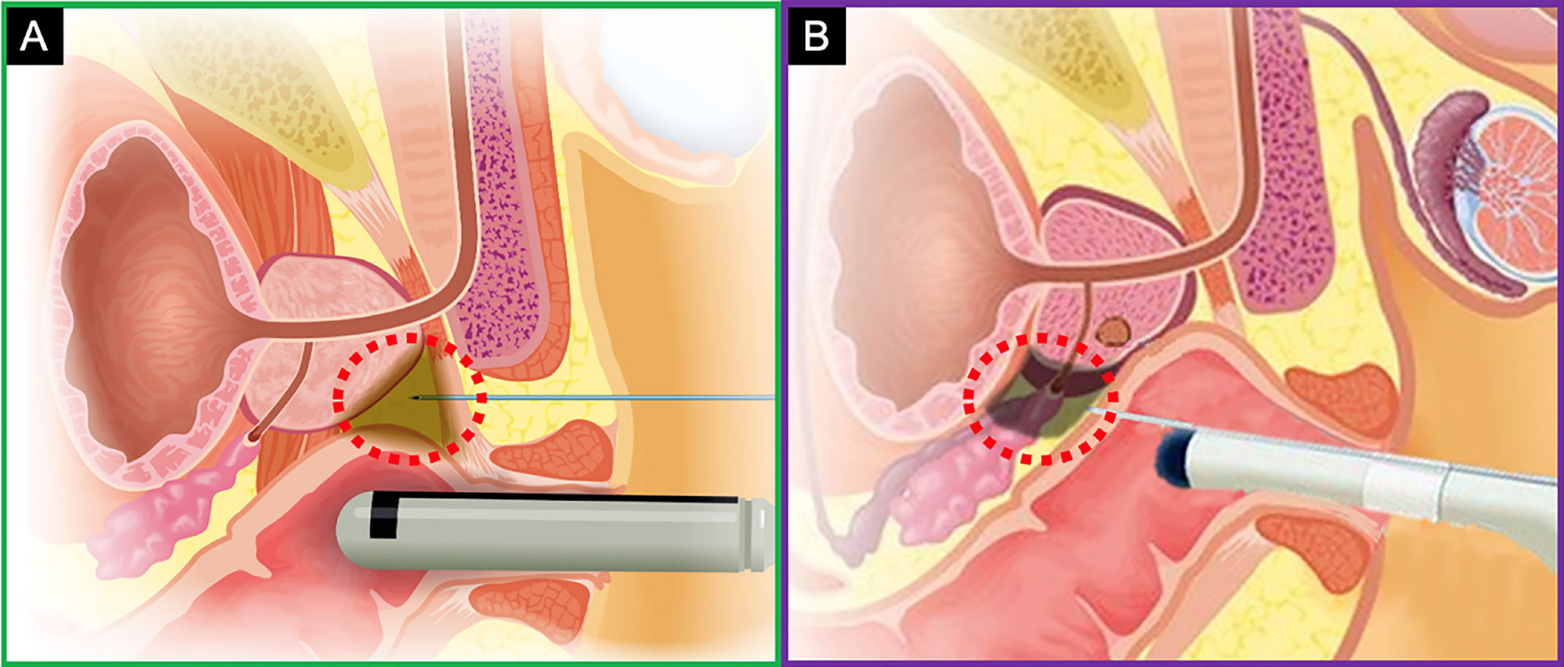
A) Periapical triangle nerve block – performed during transperineal prostate biopsy. B) Periprostatic nerve block at the junction of the prostate base, seminal vesicles, and bilateral neurovascular bundles – performed during transrectal biopsy

For a TP biopsy, the patient is placed into dorsal lithotomy position. A total of 10 mL of 2% lidocaine gel is instilled into the rectum and a digital rectal exam is performed. The perineum is prepped with chlorhexidine and the patient is draped. A side-fire endocavity 3D-TRUS probe is then inserted into the rectum. The local anesthesia mixture consists of 40mL of 0.5% lidocaine, as follows: 20 mL of Lidocaine 1%, 18mL of NaCl 0.9% (normal saline) and 2mL of sodium bicarbonate 18%. Sodium bicarbonate is added to the mixture to increase the pH of the acidic anesthetic solution which help reduce a burning sensation. Overall, 5mL of the solution is injected into the perineal skin of each side about 1-2cm anterior to the rectum and 1-2cm lateral to the midline (Figure-1) (14). Under real-time TRUS guidance, a periapical triangle (bounded by levator ani, rhabdosphincter and external anal sphincter muscles) block is then performed using an 18-G spinal needle inserted through a 17-G coaxial introducer needle with 15mL of 0.5% lidocaine used on each side. The local anesthesia is injected into the perineum and periprostatic tissue as the needle is advanced towards the prostatic apex. The 18-G spinal needle is carefully removed, and the co-axial needle sheath is left in place.

For a TR biopsy, the patient is placed into left lateral decubitus position. A total of 10 mL of 2% lidocaine gel is instilled into the rectum and a digital rectal exam is performed. An end-fire endocavity 3D-TRUS probe is then inserted into the rectum. Local anesthesia was administered via a periprostatic nerve block, under real-time TRUS guidance, injecting 10mL of 1% lidocaine solution, 5mL in each side, at the bilateral neurovascular bundles at the junction of the prostate with the seminal vesicles.

### Race, Ethnicity and Socioeconomic Status

Race and ethnicity were self-reported according to NIH standards and categorized as follows: Hispanic/Latino (Latino), non-Hispanic Asian (Asian), non-Hispanic Black or African American (Black), non-Hispanic White (White), and Others, which included individuals who did not report or identify with any specific race or ethnicity (19). Socioeconomic status (SES) was assessed using the Distressed Community Index (DCI) (database available at https://eig.org/distressed-communities), a metric developed by the Economic Innovation Group to evaluate the economic well-being of U.S. communities. The DCI is derived from the Census Bureau's Business Patterns and the American Community Survey 5-Year Estimates (2016–2020) and provides a zip code-based composite score. This score incorporates community education levels, poverty rate, unemployment rate, housing vacancy rate, median household income, as well as changes in employment and business establishments. The DCI score ranges from 0 to 100, where 0 represents the most prosperous communities and 100 indicates the most economically distressed. Higher DCI scores correspond to lower SES (20). Each patient's 5-digit zip code was matched to the DCI database, and their respective DCI scores were analyzed.

### Endpoints

The primary endpoint was pain. The patients’ self-assessed pain during PBx, using a visual analog scale ranging from 0 to 10, was assessed immediately after the procedure. Patients were asked to rate the overall pain experienced during the biopsy with the Wong-Baker FACES Pain Scale (Supplemental Figure). The FACES scale is recommended for people ages three and older and is a self-assessment tool for physical pain. The tool comes with instructions which include explaining to the patient the amount of pain each face depicts.

Secondary endpoints included PCa and CSPCa detection rates and procedure time (recorded from the moment the TRUS was inserted into the patient's rectum to the moment it was removed).

### Statistical Analysis

The Wilcoxon rank sum test was used for continuous variables, and Pearson's chi-square or Fisher exact test was used for categorical variables. Univariable and multivariable linear regression analyses were performed to correlate clinical parameters related to pain. Statistical analyses were conducted using SAS version 9.4 (SAS Institute Inc., Cary, NC, USA). A two-sided p-value of <0.05 was considered statistically significant.

## RESULTS

A total of 419 patients underwent TP (n= 322, 77%) or TR (n= 97, 23%) PBx. Overall, 14% of patients were Asian, 5% Black, 17% Latino, 12% Others, and 53% White (Table-1). The median age, PSA, PSA density, prostate volume, PIRADS distribution, PBx approach (TP or TR), and the number of cores sampled were similar between ethnical/racial groups. Clinically significant prostate cancer was detected in 60.1% (196/326) of patients with PIRADS 3-5 lesions and 11.8% (11/93) of patients with PIRADS 1-2 lesions. There was no significant difference for detection of CSPCa based on PIRADS scores between ethnic groups (Table-2A).

**Table 1:** Baseline characteristics subdivided by patient race / ethnicity.

		All	Asian	Black	Latino	Other	White	p
**No. of Patients, n (%)**		419	58 (13.8)	21 (5)	72 (17.2)	48 (11.5)	220 (52.5)	
**Age, year, mean (SD)**		66.3 (8.0)	67.6 (9.6)	66.8 (7.4)	65 (8.6)	67.2 (6.6)	66.1 (7.7)	0.21
**BMI, mean (SD)**		28.4 (9.7)	25.1 (3.5)	27.5 (6.7)	30.2 (4.1)	28.4 (4.3)	27.9 (4.0)	<.0001
**Family History PCa, n (%)**		108	11 (19.6)	6 (30)	12 (18.8)	9 (20.5)	70 (33.8)	0.05
**Biopsy History, n (%)**								0.11
	Naïve	272	38 (65.5)	17 (81)	38 (53.5)	26 (56.5)	153 (69.9)	
	Negative	73	11 (19)	1 (4.8)	16 (22.5)	13 (28.3)	32 (14.6)	
	In active surveillance	70	9 (15.5)	3 (14.3)	17 (23.9)	7 (15.2)	34 (15.5)	
**PSA, ng/mL, mean (SD)**		9.8 (18.8)	7.4 (3.6)	8.5 (7.5)	12.4 (27.8)	10.0 (13.1)	9.7 (19.3)	0.97
**PSA density, ng/mL^2^, mean (SD)**		0.2 (0.4)	0.2 (0.1)	0.2 (0.2)	0.2 (0.5)	0.2 (0.4)	0.2 (0.4)	0.69
**Suspicion for PCa on DRE, n (%)**		98	11 (19)	3 (14.3)	18 (25)	11 (22.9)	55 (25)	0.73
**PIRADS score, n (%)**								0.36
	PIRADS 1-2	93 (22.2)	17 (29.3)	7 (33.3)	15 (20.8)	8 (16.7)	46 (20.9)	
	PIRADS 3-5	326 (77.8)	41 (70.7)	14 (66.7)	57 (79.2)	40 (83.3)	174 (79.1)	
**Prostate volume, mean (SD)**		59.1 (15.3)	52.3 (24.2)	62.4 (51.6)	67.6 (38.7)	59.6 (35.2)	57.6 (29.3)	0.16
**No. of MRI lesions** **mean (SD)**		1.1 (0.8)	1.1 (0.8)	0.8 (0.7)	1.2 (0.9)	1.2 (0.7)	1.1 (0.8)	0.53
**MRI index lesion location, n (%)**								
	Anterior	136	16 (27.6)	5 (23.8)	22 (30.6)	26 (54.2)	67 (30.5)	**0.01**
	Posterior	247	28 (48.3)	12 (57.1)	42 (58.3)	24 (50)	141 (64.1)	0.15
**MRI index lesion location, n (%)**								
	Base	98	11 (19)	3 (14.3)	20 (27.8)	9 (19)	55 (25)	0.51
	Mid	222	33 (56.9)	10 (47.6)	37 (51.4)	28 (58.3)	114 (51.8)	0.85
	Apex	117	16 (27.6)	3 (14.3)	18 (25)	19 (39.6)	61 (27.7)	0.24
**MRI index lesion size, mm, mean (SD)**		15.3 (7.5)	13.4 (5.9)	13.9 (5.0)	17.0 (8.8)	15.2 (8.1)	15.3 (7.4)	0.37
**No. of (SB + TB) cores taken mean (SD)**		15.0 (2.2)	15.1 (2.6)	14.2 (2.5)	15.4 (1.6)	14.7 (2.6)	15 (2.1)	0.22
**No. of TB cores taken** **mean (SD)**		3.9 (2.9)	3.7 (2.8)	3.0 (2.5)	3.8 (2.8)	4.0 (2.4)	4.1 (3.0)	0.5
**Biopsy approach, n (%)**								0.63
	Transperineal	322 (77)	47 (81)	15 (71.4)	51 (70.8)	37 (77.1)	172 (78.2)	
	Transrectal	97 (23)	11 (19)	6 (28.6)	21 (29.2)	11 (22.9)	48 (21.8)	
**Distress Community Index Quintiles, n (%)**								**<.0001**
	Prosperous (0-20)	133	17 (29.8)	7 (35.0)	9 (12.9)	16 (34.0)	84 (39.1)	
	Comfortable (20-40)	94	14 (24.6)	3 (15)	10 (14.3)	11 (23.4)	56 (26.1)	
	Mid-tier (40-60)	83	18 (31.6)	4 (20.0)	6 (8.6)	11 (23.4)	44 (20.5)	
	At risk (60-80)	60	6 (10.5)	2 (10.0)	26 (37.1)	5 (10.6)	21 (9.8)	
	Distressed (80-100)	39	2 (3.5)	4 (20.0)	19 (27.1)	4 (8.5)	10 (4.7)	

BMI = body mass index; DRE = digital rectal examination; IQR = interquartile range; MRI = magnetic resonance imaging; No. = number; PCa = prostate cancer; PIRADS = Prostate Imaging Reporting and Data System; PSA = prostate specific antigen; SB = standard biopsy; SD = standard deviation; TB = target biopsy

**Table 2A:** Pathologic Outcomes of MRI/TRUS fusion prostate biopsy.

	PIRADS 1-2						PIRADS 3-5					
	Asian	Black	Latino	Other	White	p	Asian	Black	Latino	Other	White	p
No. of Patients, n (%)	17	7	15	8	46		41	14	57	40	174	
PCa detection SB + TB, N (%)	4 (23.5)	4 (57.1)	7 (46.7)	2 (25)	19 (41.3)	0.43	32 (78.1)	11 (78.6)	35 (61.4)	32 (80)	130 (74.7)	0.20
PCa detection SB, N (%)	4 (23.5)	4 (57.1)	7 (46.7)	2 (25)	19 (41.3)	0.43	24 (58.5)	10 (71.4)	30 (52.6)	22 (55)	102 (58.6)	0.76
PCa detection TB, N (%)	-	-	-	-	-	-	28 (68.3)	11 (78.6)	31 (54.4)	28 (70)	117 (67.2)	0.30
CSPCa SB + TB, N (%)	2 (11.8)	1 (14.3)	4 (26.7)	1 (12.5)	3 (6.5)	0.35	28 (68.3)	10 (71.4)	32 (56.1)	23 (57.5)	103 (59.2)	0.65
CSPCa SB, N (%)	2 (11.8)	1 (14.3)	4 (26.7)	1 (12.5)	3 (6.5)	0.35	15 (36.6)	8 (57.1)	22 (38.6)	16 (40)	68 (39.1)	0.73
CSPCa TB, N (%)	-	-	-	-	-	-	25 (61)	9 (64.3)	28 (49.1)	21 (52.5)	97 (55.8)	0.73

CSPCa = clinically significant prostate cancer; No. = number; PCa = prostate cancer; PIRADS = Prostate Imaging Reporting and Data System; SB = systematic biopsy; TB = target biopsy

**Figure 2:**
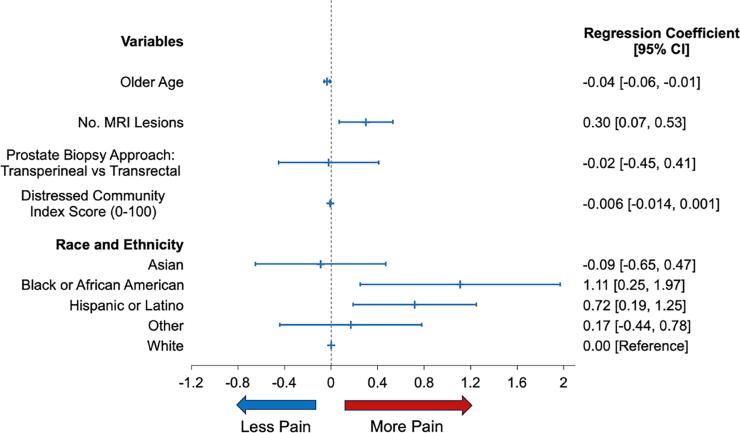
Patients’ self-assessed pain level during transperineal or transrectal prostate biopsy under local anesthesia

**Figure 3:**
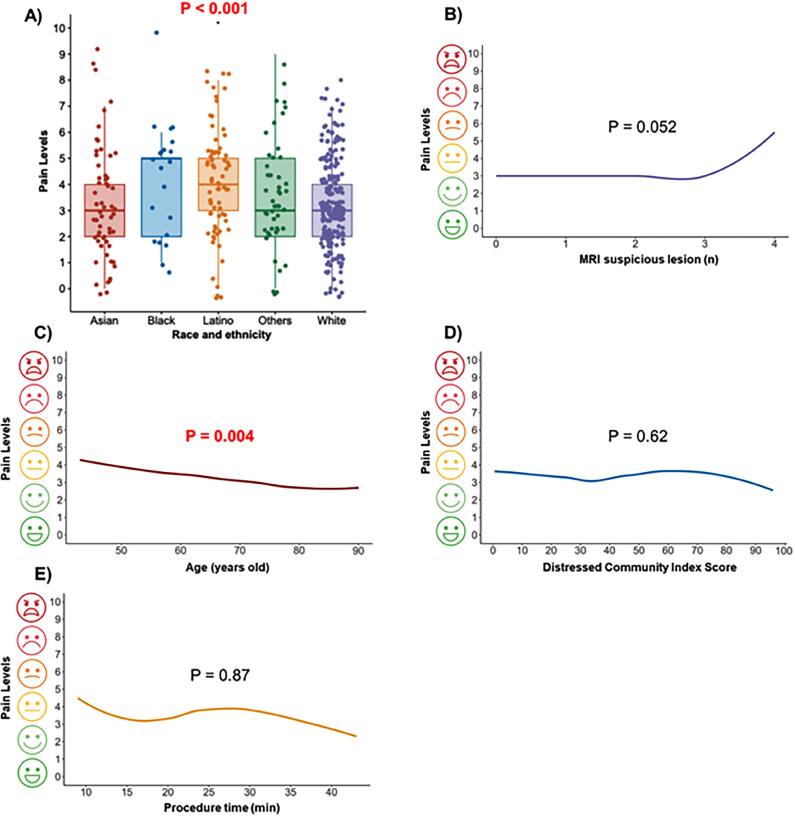
Distribution of Pain Levels (A). Reported pain levels by Number of MRI Suspicious Lesions (B), Age (C), DCI (D), Procedure Time (E)

Of the cohort, 20% of Black and 27% of Latino patients were most distressed (DCI 80-100), in contrast to 4% of Asian, 9% of Other, and 5% of White patients (p<0.001) (Table-1). The median (IQR) self-assessed pain level for all patients was 3 (2-5). Pain levels were higher for Black 5 (2-5) and Latino 4 (3-5) compared to Asian 3 (2-4), Other 3 (2-5), and White 3 (2-4) patients (p=0.01) (Table-2B, Figures 2 and 3). Median operative time was 15 minutes (IQR 15 – 21 minutes), and it was not significantly different between ethnic groups (Tables 1 and 3, Figures 2 and 3). Pain levels were not statistically significantly associated with increased procedure time (β 0.003, 95% CI −0.03, 0.04; p = 0.87) or the detection of prostate cancer (β −0.11, 95% CI −0.51, 0.28; p = 0.57). On multivariable analysis, younger age (β −0.04, 95% CI −0.06, 0.01), Black (β 1.11, 95% CI 0.25, 1.98) or Latino (β 0.72, 95% CI 0.19, 1.26) race, and the number of lesions on MRI (β 0.30, 95% CI 0.07, 0.53) were independent predictors for pain levels, but the biopsy approach (TP or TR) and the DCI were not (Table-3, Figure 2 and 3).

Each graph represents the smoothed median pain scale across age, DCI, procedure time, or number of MRI-suspicious lesions, fitted using LOESS regression to highlight the general trend in pain scale variation with each category. P value of MRI suspicious lesion was not statistically significant (p=0.052) on univariable analysis but significant (p=0.008) on multivariable analysis.

**Table 2B:** Perioperative outcomes after MRI/TRUS fusion prostate biopsy.

	Race/Ethnic Groups						
	All	White	Black	Latino	Asian	Other	p
**Number of patients, n (%)**	419	220 (52.5)	21 (5)	72 (17.2)	58 (13.8)	48 (11.5)	
**Pain during biopsy, median (IQR)**	3 (2-5)	3 (2-4)	5 (2-5)	4 (3-5)	3 (2-4)	3 (2-5)	0.01
**Procedure time for PBx, minutes, median (IQR)**	18 (15-21)	18 (16-20)	17 (15-21)	18 (15-20)	18 (14-22)	20 (17-25)	0.06

IQR = interquartile range; PBx = prostate biopsy

**Table 3:** Univariable and Multivariable analyses for pain level during prostate biopsy under local anesthesia.

Variables		Univariate			Multivariate		
		β	CI (95%)	p	β	CI (95%)	p
**Age, year**		-0.03	-0.06 to −0.01	**0.004**	-0.03	-0.06 to −0.01	**0.006**
**Family History PCa**		-0.49	-0.92 to −0.07	**0.02**			
**BMI**		-0.01	-0.03 to 0.01	0.40			
**Biopsy history**							
	Previous Negative biopsy vs Naïve	-0.14	-0.65 to 0.37	0.58			
	Previous Positive biopsy vs Naïve	0.17	-0.34 to 0.69	0.50			
**PSA, ng/mL**		-0.003	-0.013 to 0.006	0.52			
**PSA density** * **, ng/mL^2^**		-0.11	-0.57 to 0.36	0.65			
**Race**							
	Asian vs NH-White	-0.05	-0.61 to 0.51	0.85	-0.11	-0.68 to 0.45	0.69
	Hispanic vs HN-White	0.77	0.26 to 1.29	**0.003**	0.87	0.30 to 1.45	**0.003**
	Black vs NH-White	0.94	0.07 to 1.81	**0.03**	1.16	0.29 to 2.03	**0.009**
	Others vs NH-White	0.27	-0.34 to 0.87	0.39	0.17	-0.45 to 0.79	0.59
**DRE, suspicious vs non-suspicious**		0.12	-0.32 to 0.56	0.59			
**Prostate Volume, cc**		-0.005	-0.010 to 0.001	0.11			
**No. MRI lesions**		0.23	-0.002 to 0.462	0.052	0.32	0.08 to 0.55	**0.008**
**MRI lesion size, mm**		0.006	-0.023 to 0.034	0.69			
**PIRADS 3-5 vs PIRADS 1-2**		0.31	-0.14 to 0.76	0.18			
**No. TB cores taken**		-0.04	-0.12 to 0.05	0.40			
**Prostate biopsy approach TP vs TR**		0.03	-0.04 to 0.10	0.37	0.01	-0.43 to 0.45	0.95
**No. Bx (SB + TB) cores taken**		-0.16	-0.60 to 0.29	0.49			
**Procedure Time (min)**		0.003	-0.033 to 0.039	0.16			
**Presence of Prostate Cancer**		-0.11	-0.51 to 0.28	0.57			
**Distress Community Index Score (0-100)**		-0.001	-0.009 to 0.005	0.62	-0.006	-0.014 to 0.001	0.10

PIRADS = Prostate Imaging Reporting and Data System; MRI = magnetic resonance imaging; OR = odds ratio; CI = confidence interval; PCa = prostate cancer; CSPCa = Clinically significant PCa (Grade Group > 1); DRE = digital rectal examination; DRE = digital rectal examination; NH = non-Hispanic

* PSA density was calculated per 0.01 unit.

## DISCUSSION

There were 419 patients in our cohort who underwent prostate biopsy with a transperineal or transrectal approach. There was no significant difference in pain based on approach. The median pain level for all patients was 3 / 10, and it was significantly higher for Black patients and Latino patients. Other predictors of pain included younger age and increased number of lesions on MRI. There was no association of pain with prostate size, biopsy history, operative time or PIRADS score on univariable analysis or DCI on multivariable analysis.

Our study is the first to demonstrate an ethnic difference in pain during prostate biopsy. While this has not been shown before in the setting of prostate biopsy, there have been other studies which have exhibited that Latino and Black patients have increased pain perception. Perry et al. performed a systematic review of a variety of procedures including tonsillectomy, hip and knee arthroplasty demonstrating that there were significantly higher preoperative and postoperative pain intensity scores reported by African American and Hispanic individuals compared with non-Hispanic whites (21). Meanwhile, in the laboratory setting, Hastie et al. experimented on thermal, pressure, and ischemic pain perception between non-Hispanic whites, non-white Hispanics and African Americans, and demonstrated that African Americans and Hispanics had decreased pain tolerance (22).

The reason for difference in pain perception in ethnicity is unclear, but it is likely multifactorial. One possible reason for increased pain in certain ethnicities is the presence of different genetic predispositions. In the review by Perry et al., they reported on studies showing that polymorphisms in the genes Catechol-O-methyltransferase (COMT) and mu opioid receptor 1 (OPRM1) were associated with pain. Specifically, there is a single nucleotide polymorphism (SNP) in codon 158 (val158met) which affects COMT protein stability and has been associated with pain (23). Lee et al. demonstrated that COMT polymorphisms associated with high pain sensitivity were more prevalent in African Americans than Caucasian patients (24). Meanwhile Hastie et al. demonstrated that a specific polymorphism for OPRM1 was present in about 28% of Hispanic patients and it was associated with increased pain sensitivity for Hispanic patients but not for Caucasian patients (22). While no study has looked specifically at genetic predispositions to prostate biopsy pain, there is evidence that genetic polymorphisms exist associated with pain.

Socioeconomic status has also been reported to be associated with increased postoperative pain. Additionally, it has been reported that there are inequalities in SES strongly patterned by race including African Americans persistently having higher levels of poverty than Caucasian people (25). Furthermore, lower SES has been shown to be associated with poorer mental health and increased psychologic stress which may impact pain perception (26). Thurston et al. found in a systematic review that lower SES was associated with worse pain perception after various procedures (8). Pain was not associated with DCI in our study, which could be related to its limited ability to accurately assess an individual patient's SES given that it is based on location (zip code).

Our study also demonstrated that pain was associated with younger age Younger age was also associated with prostate biopsy pain in studies by Marra et al. and Gomez-Gomez et al. (27, 28). As younger patients have had less exposure to health care and different medical interventions, increased pain perception may be attributed to a decreased familiarity with pain from such procedures (27, 28). We additionally demonstrated increased pain scores with a higher number of MRI lesions, despite number of biopsy cores, prostate size, operative time and lesion size not being significant. Upon examining Figure-3 the increased pain was seen in those with four different target lesions. It is possible that excessive manipulation of the needle and probe could lead to more discomfort and increase patient anxiety.

We have identified several patient characteristics associated with increased pain during biopsy: younger age, Latino ethnicity, Black race and multiple MRI lesions. Recognizing populations who are at more risk of severe pain is important as such patients can be counseled to undergo a biopsy under sedation. Cricco-Lizza et al. demonstrated that transperineal prostate biopsies under local anesthesia is safe and has comparable outcomes compared to those done under sedation, but median pain scores were 3/10 versus 0/10, respectively (29). Escobar et al. also demonstrated significant decrease in pain scores during transrectal prostate biopsy performed with the use of nitrous oxide in a randomized trial (30). Pre-procedure counseling regarding higher pain levels and discussion of additional strategies to mitigate pain can be performed in these populations with increased pain risk.

There are some limitations of our study. First, race and ethnicity can be broken down further to specific countries where patients’ families are from which can further impact genetic and cultural factors. Second, DCI uses zip codes to determine SES at a community-level and is therefore limited in its ability to accurately identify SES at an individual level for each patient. Meanwhile, strengths include the diversity of the patient population and that the data is reliable given that it was collected prospectively through a well-managed protocol in a center of extensive experience in both transperineal and transrectal prostate biopsy (13–15).

## CONCLUSIONS

We demonstrate that the risk factors for higher pain levels during prostate biopsy under local anesthesia are Black and Latino race, younger age and increased number of MRI lesions. Meanwhile, socioeconomic status did not have an impact on pain levels. Upon counseling patients for prostate biopsy, risk factors of higher pain levels may be discussed to ensure they are well-informed when making the decision to have a biopsy performed under local anesthesia versus sedation. Further validation studies are warranted.

## Data Availability

All data generated or analysed during this study are included in this published article

## ABBREVIATIONS

PBx: = Prostate Biopsy
LA: = Local Anesthesia
TP: = Transperineal
TR: = Transrectal
DCI: = Distressed Community Index
SES: = Socioeconomic Status
mpMRI: = Multiparametric MRI
PIRADS: = Prostate Imaging Reporting & Data System
CSPCa: = Clinically Significant Prostate Cancer
