# Supplementary material for: Pain Perception During Transperineal and Transrectal Prostate Biopsy Under Local Anesthesia: a Prospective Analysis of a Multi-ethnic and Diverse Cohort
Source: Int Braz J Urol. 2026 Jan 28;52(3):e20250512. doi: 10.1590/S1677-5538.IBJU.2025.0512 (PMC13124175; doi:10.1590/S1677-5538.IBJU.2025.0512)

APPENDIX

Supplemental Figure - Wong-Baker FACES Pain Rating Scale.

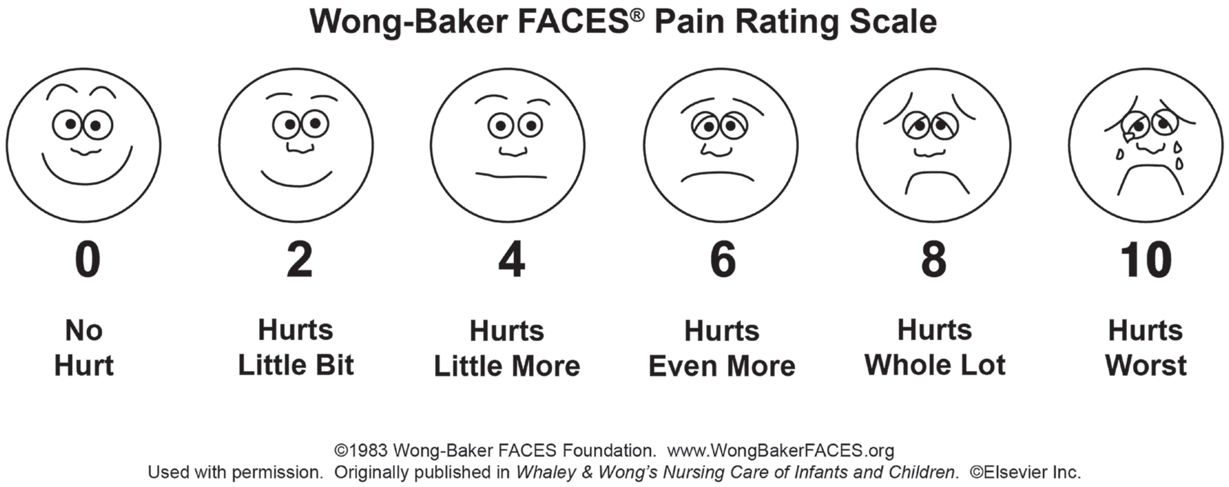

Supplement: APPENDIX [file 1677-6119-ibju-52-03-e20250512-suppl1.pdf]
